# Construction Notes

**Published:** 1894-12-08

**Authors:** 


### CONSTRUCTION NOTES.

### New Wing, Heatherdene Convalescent Home.

The second anniversary of the opening of Heatherdene Convalescent Home, Harrogate (an adjunct of the Sunderland Infirmary, which this year is celebrating its centenary), was commemorated by the laying of the foundation-stone of a new wing to the home. The total cost of this, including furnishing and an extra two acres of land, is £4,500, and towards this the committee have in hand a promised £2,700. The extension will be three storeys high, the ground floor being devoted to the administrative part of the building. The wards for the patients will be on the first and second floors. A day-room is also provided for the use of male patients. Adjoining the dining-room is a kitchen, and in direct communication with this are the other essential offices. The new wing will be erected entirely of stone, and in complete harmony with the existing building. It is estimated that this will be ready for occupation and completed in twelve months. It is designed by Mr. John Eltringham, architect, of Sunderland, and the contractors are Messrs. Ives and Co., Shipley.

### New Wing Croydon General Hospital.

This addition, recently made, consists of a three-storeyed building, containing on the ground floor a ward of 86 feet long, having a cubic capacity of about 31,500 feet. It will hold 20 beds, which will be used for men patients. In an annexe are bath-room, lavatory, scullery, and other conveniences. The ward is connected with the old building by a corridor eight feet wide. On one side of this is a nurse's room overlooking the ward and a ward kitchen. On the other side of this is a store for the matron and an exit to the grounds. The first floor is a replica of the ground floor, except that the two items last named do not recur. The ward on this floor will be used for women. On the second floor is a large kitchen, with sculleries, larder, pantries, stores, and also a room for airing linen, a sewing-room, and a sitting-room for nurses. Coal and dinner lifts are provided to each floor. In a spacious basement are coal and coke cellars, also heating apparatus capable of comfortably warming the corridors, wards, &c. Fireplaces are also provided in the wards and other rooms in case of any excessively cold weather. Careful attention has been paid to the ventilation. Fresh air will be admitted through the tubular hot-water radiators—cool in summer and warm in winter. For the extraction of spent and vitiated air suitable flues are carried up to turrets on the roof. These turrets give character and pleasing variety of outline to a building necessarily otherwise plain and severe in external appearances. The sanitary fittings are of the most modern and approved description, and the drainage has been very carefully executed. Between the visible ceilings and the floors exists steel and concrete construction, making the building absolutely fireproof. The contractor was Mr. Samuel Page, who worked from the designs of Mr. Charles Henman, A.R.I.B.A.

